# Metabolic‐immune interactions in gastric cancer T cells: A single‐cell atlas for prognostic biomarker identification

**DOI:** 10.1002/qub2.70027

**Published:** 2026-01-01

**Authors:** Junjun Liu, Rui Zhao, Guodong Yao, Zhao Liu, Runze Shi, Jingshu Geng, Guanying Liang, Kexin Chen

**Affiliations:** ^1^ Department of Pathology Harbin Medical University Cancer Hospital Harbin China; ^2^ Department of Otolaryngology‐Head and Neck Surgery The Second Affiliated Hospital of Harbin Medical University Harbin China; ^3^ Department of Ultrasound Harbin Medical University Cancer Hospital Harbin China; ^4^ Department of Breast Surgery Harbin Medical University Cancer Hospital Harbin China

**Keywords:** gastric cancer, metabolic phenotypes, prognostic biomarkers, single‐cell RNA‐sequencing, T cells

## Abstract

Metabolic alterations and immune dysfunction within the gastric tumor microenvironment critically drive gastric cancer (GC) progression and therapeutic resistance. Although single‐cell RNA sequencing (scRNA‐seq) has unveiled cellular heterogeneity in GC, the metabolic landscapes of tumor cells and their interplay with immune components remain underexplored. By integrating scRNA‐seq data from 35,633 cells across 23 GC tissues (GSE150290), bulk RNA‐seq data from UCSC Xena, and two independent microarray cohorts (GSE26899, GSE62254), we systematically characterized metabolic heterogeneity and identified immune‐related prognostic biomarkers. Reclustering of malignant epithelial cells revealed distinct metabolic phenotypes, with the citrate cycle and oxidative phosphorylation pathways emerging as key drivers of intratumoral diversity and T cell differentiation. Through machine learning and survival analyses, we discovered a novel risk score model composed of 6 T cell differentiation signatures, which stratified patients into high‐ and low‐risk groups with significant differences in overall survival. Notably, this model outperformed traditional clinicopathological factors in predicting prognosis, validated in both bulk RNA‐seq and microarray datasets. Immunohistochemistry further confirmed the prognostic value of key regulatory proteins (RGS1, CXCR4, CTLA4, ARPP19, ZNRF1, and ZNF207). Our findings highlight the metabolic immune crosstalk in GC and provide a promising biomarker panel for precision risk stratification and potential immunotherapeutic targets.

## 1 INTRODUCTION

Gastric cancer (GC) continues to represent a significant health challenge globally, consistently listed as one of the most common causes of cancer‐related death worldwide [1]. Despite high curability in early stages, over 70% of patients present with advanced disease at diagnosis, resulting in poor survival outcomes [2, 3]. In 2022, GC caused an estimated 660,000 deaths worldwide [4]. Although immune checkpoint blockade has revolutionized cancer immunotherapy across multiple malignancies [5], its efficacy in GC remains limited, with response rates below 20% in unselected cohorts [6]. Although extensive research has characterized molecular heterogeneity within the GC tumor microenvironment (TME), translating these findings into clinical applications has proven challenging. Consequently, integrating single‐cell resolution analyses of malignant cell biology with immune dysregulation mechanisms represents a critical path toward precision therapeutics.

Tumor initiation and progression necessitate the metabolic reprogramming of cancer cells to fulfill their heightened requirements for energy, biomass, and cellular communication [7, 8]. Cancer cells adapt their metabolism to fulfill increased energy and biosynthesis requirements, while also managing oxidative stress, thereby supporting their growth and survival [9, 10]. The metabolic activity within cells is contingent upon the concentration of metabolically significant molecules and the rate of biomolecule conversion; however, these parameters can be challenging to measure directly. Hence, it becomes crucial to access the expression of metabolic genes as an indirect approach to gauge the metabolic activity of cells.

A tumor is not merely a cluster of cancer cells; rather, it is a complex and diverse assembly of infiltrating and resident host cells, secreted factors, and extracellular matrix [11, 12]. Malignant cells actively reshape their local tissue microenvironment at molecular, cellular, and biomechanical levels, promoting tumor progression and growth [13]. Within this altered landscape, different cell populations possess unique metabolic profiles, reflecting their distinct roles and evolutionary trajectories, which delineate tumor cells from their non‐malignant counterparts [14]. Notably, among the diverse immune cells infiltrating the TME, lymphoid T cells are pivotal in modulating immune responses and mediating tumor cell eradication [15, 16]. CD8^+^ cytotoxic T cells and CD4^+^ helper T cells, the primary T cell subsets, play distinct roles in anti‐tumor immune responses. However, the specific mechanisms regulating their differentiation are not yet fully understood.

In this study, we analyzed single‐cell expression profiles from 23 GC samples to identify metabolic pathways associated with tumor cell heterogeneity and T cell differentiation at single‐cell resolution. Furthermore, we developed a novel risk score model that shows potential as a prognostic biomarker.

## 2 RESULTS

**FIGURE 1:**
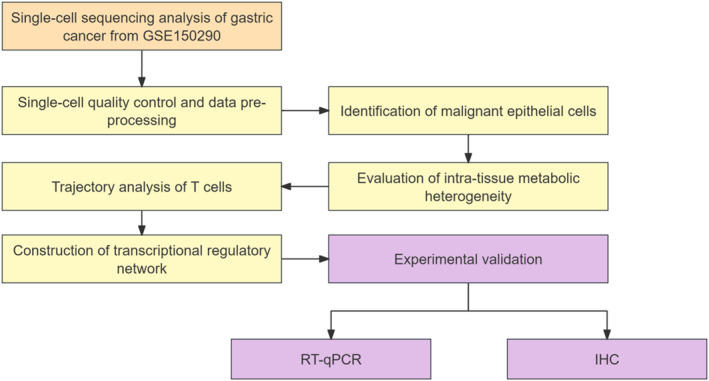
Overview of the study design.

This study delineates a comprehensive analytical workflow to investigate the TME of GC using single‐cell RNA sequencing (scRNA‐seq) data from the GSE150290 dataset. The research commenced with single‐cell quality control and data pre‐processing to ensure data integrity. Subsequently, malignant epithelial cells were accurately identified and isolated for further scrutiny. The investigation then proceeded to concurrently assess intra‐tissue metabolic heterogeneity and perform trajectory analysis of T cells to delineate both metabolic and functional T cell dynamics. Furthermore, a transcriptional regulatory network was constructed to unravel the underlying gene regulatory mechanisms. Finally, key findings from the bioinformatic analyses were subjected to experimental validation using RT‐qPCR and immunohistochemistry (IHC) to confirm their biological and pathological relevance (Figure 1).

### 2.1 Single‐cell transcriptional profiling in GC patients

**FIGURE 2:**
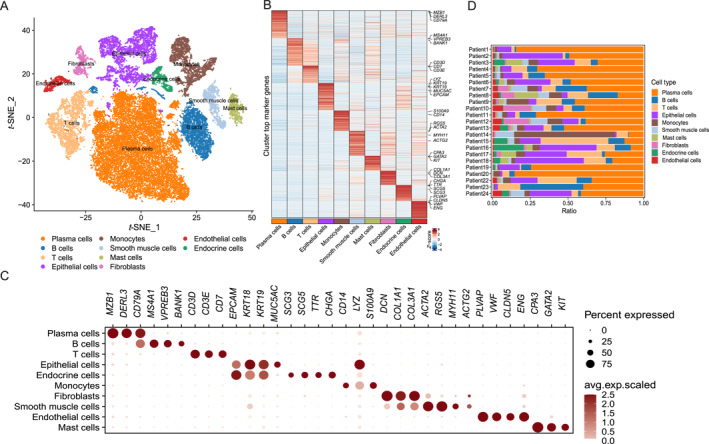
Single‐cell transcriptomic profiling reveals distinct T cell subsets in the gastric cancer tumor microenvironment. (A) *t*‐distribution stochastic neighbor embedding plot visualizing all identified cell subpopulations. (B) Heatmap showing the scaled average expression of the top 10 marker genes for each cluster. (C) Dot plot displaying expression of key marker genes across T cell clusters. Dot size indicates the percentage of cells expressing the gene, whereas color denotes the average expression level. (D) Bar chart quantifying the proportion of cells from each sample origin within the major cell types.

To elucidate the cellular heterogeneity of GC at single‐cell resolution, we integrated expression profiles across 23 GC tissues and retained 35,633 cells after quality control and filtering procedures. To discern distinct cellular subpopulations based on gene expression profiles, we employed dimensionality reduction and unsupervised cell clustering using methods implemented in the Seurat software. As shown by *t*‐distribution stochastic neighbor embedding, we clustered all cells and divided them into 23 clusters. Based on the expression of known markers, we finally annotated 10 cell types spanning epithelial cells (*EPCAM*, *KRT18*, *KRT19*, *MUC5AC*), monocytes (*CD14*, *LYZ*, *S100A9*), fibroblasts (*DCN*, *COL1A1*, *COL3A1*), plasma cells (*CD79A*, *MZB1*, *DERL3*), B cells (*CD79A*, *BANK1*, *VPREB3*, *MS4A1*), T cells (*CD3E*, *CD3D*, *CD7*), smooth muscle cells (*ACTA2*, *RGS5*, *MYH11*, *ACTG2*), endocrine cells (*SCG3*, *SCG5*, *TTR*, *CHGA*), endothelial cells (*PLVAP*, *VWF*, *CLDN5*, *ENG*), and mast cells (*CPA3*, *GATA2*, *KIT*) (Figure 2A–C). Each type of cell could be found in all patients, and the distribution was different. Patients 15 and 22 exhibited higher T cell infiltration, which is consistent with an immune‐inflamed TME previously associated with improved response to immunotherapy in some cancers. However, this observation remains hypothetical in the absence of validated biomarkers such as PD‐L1 expression, microsatellite instability, or tumor mutational burden, and thus requires further validation (Figure 2D).

### 2.2 Metabolic heterogeneity defines malignant cell states in GC

**FIGURE 3:**
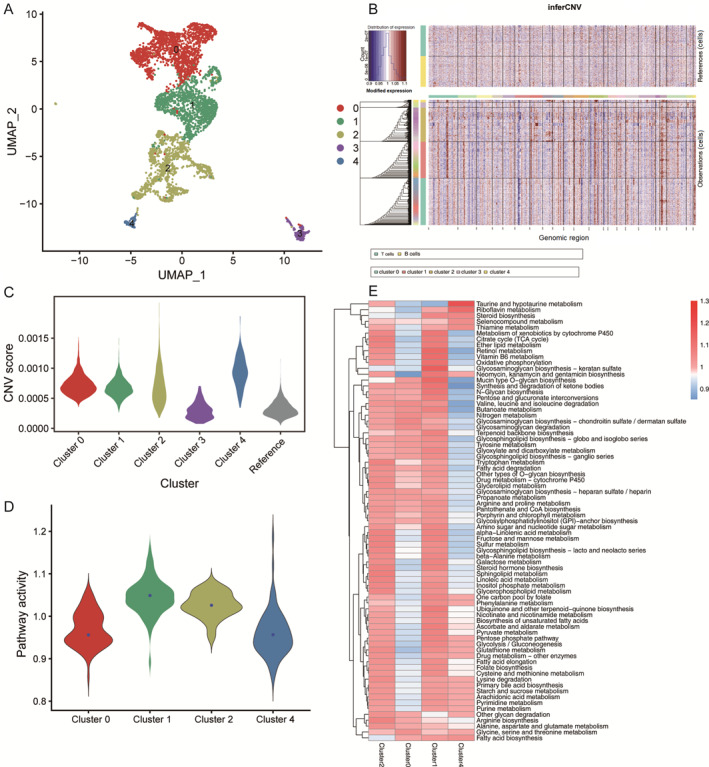
Characterizing the metabolic dependencies of gastric cancer epithelial cells. (A) Uniform manifold approximation and projection visualization of epithelial cells, highlighting distinct subpopulations through color‐coded clustering. (B) Heatmap of CNV signals normalized against the “normal” cluster shown in the top panel (T cells and B cells) for CNV changes by chromosome (columns) within individual cells (rows). (C) Violin plots display the heterogeneous CNV score profiles across epithelial cell subpopulations. (D) Distributions of pathway activities in different malignant epithelial cell clusters. (E) Metabolic pathway activity in each malignant epithelial cell cluster. Values with statistically non‐significant pathway activity (random permutation test, *p* > 0.05) were shown as blank. CNV, copy number variation.

During tumor progression and malignant transformation, the metabolic processes of different cells can be influenced by the TME, including factors such as glucose, nutrients, and oxygen availability. Consequently, we further investigated changes in metabolic features within the tumor cells. The cells identified as epithelial cells were extracted and re‐clustered, resulting in the discovery of 10 distinct clusters as shown in Figure 3A. To distinguish the tumor cells and mixed normal epithelial cells, we calculated copy number variation (CNV) scores of cells by inferring CNV, an algorithm to estimate the copy number changes in the genome with scRNA‐seq data. Finally, we identified 35,633 clusters as normal epithelial cells, as they had similar copy number variant patterns compared with normal immune cells. The result also demonstrated that despite removal of batch effects, tumor cells still displayed a high heterogeneity (Figure 3B,C).

To investigate the metabolic reprogramming change in malignant tumor cells, we employed a weighted relative pathway activity algorithm to assess the activity of metabolic‐related pathways in each cluster tumor cell. Among the 85 metabolic‐related pathways obtained from the Kyoto Encyclopedia of Genes and Genomes database, 75 pathways containing more than five genes had significantly higher pathway activity scores in at least one tumor cell cluster (pathway score >1 and permutation test *p* < 0.05) (Figure 3D). Notably, Clusters 2 and 1 exhibited an upregulated trend in many important metabolic pathways, including tricarboxylic acid cycle cycle, oxidative phosphorylation (OXPHOS), glycolysis, and tyrosine metabolism (Figure 3E). We observed significant differences in the metabolic pathway activity scores among these malignant cell clusters, suggesting that the variation in these pathways may contribute to tumor heterogeneity.

### 2.3 Exploring metabolic dynamics during T cell differentiation in GC

**FIGURE 4:**
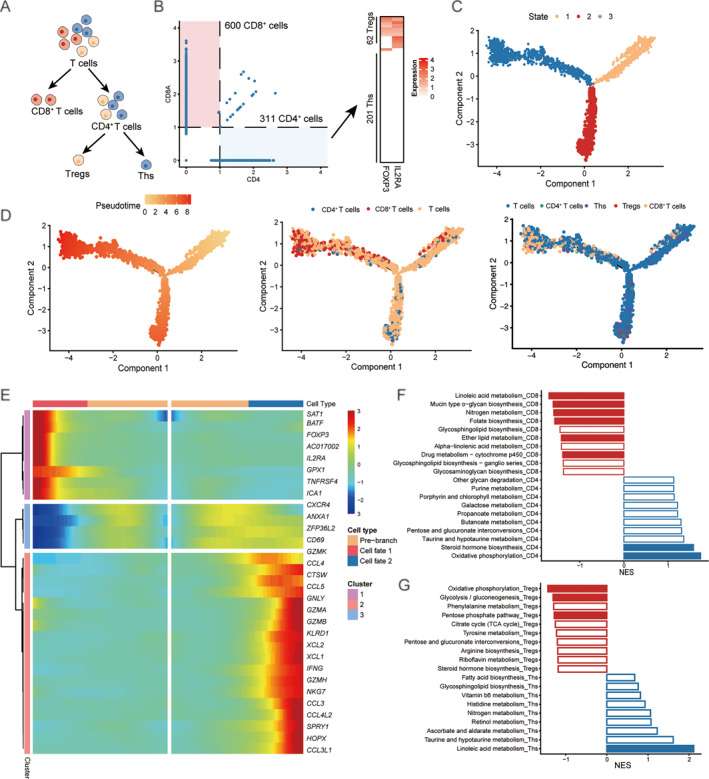
Characteristics of T cell differentiation and metabolic profiles. (A) T cell classification into distinct subtypes, including CD4^+^, CD8^+^, Tregs, and Ths. (B) Gene marker expression levels used to distinguish T cell subtypes in gastric cancer. (C) A single‐cell pseudotime trajectory depicts T cell differentiation, with each data point representing an individual cell annotated by its specific developmental stage. (D) Pseudotime trajectory visualizations showing individual cells annotated by pseudotime (left) and classified by cell subtype (middle and right). (E) The heatmap shows the branch‐dependent genes at branch point 1. The center of the heatmap is branch B1, the left is B2, and the right is B3. (F) Major metabolic pathways enriched in CD4^+^ and CD8^+^ T cells. Pathways with significant enrichment (GSEA *p* < 0.05) are highlighted in red for CD4^+^ and blue for CD8^+^ T cells. (G) Key metabolic pathways enriched in Ths and Tregs, with significant pathways (GSEA, *p* < 0.05) marked in red for Th cells and blue for Tregs. GSEA, gene set enrichment analysis; NES, normalized enrichment score.

In the TME, diverse immune cell types play distinct roles and constitute a crucial component. To understand the metabolic reprogramming of immune cells, we characterized the developmental trajectory and metabolic features of T cells, which represent the predominant immune cell population. T cells were initially separated into CD4^+^ and CD8^+^ groups based on the expression of cell markers CD4 and CD8A (Figure 4A). The CD4^+^ T cells were further classified into regulatory T cells (Tregs) and T helper cells (Ths) based on expression levels of *FOXP3* and *CD25* which are known to be specifically expressed in these specific cell types. According to the expression of these cell markers, we finally identified 600 CD8^+^ T cells and 311 CD4^+^ T cells which included 62 Tregs and 201 Ths (Figure 4B). The monocle algorithm was used to characterize the pseudo‐development trajectory of T cells, and three branches were found in the development of T cells, which may represent the three major states of T cells (Figure 4C). We further observed that branch 3 mainly included CD8^+^ T cells, while CD4^+^ T cells (including Tregs and Ths) were mainly concentrated on state 2 (Figure 4D). We identified many branch‐specific genes which were closely related to the differentiation of T cells, such as *FOXP3*, *IL2RA* and *GZMA*/*GZMB* (Figure 4E). We then performed gene set enrichment analysis (GSEA) to identify metabolic pathways enriched in each T cell subtype. The results showed that CD4^+^ T cells and CD8^+^ T cells had markedly different metabolic patterns; the CD8^+^ T cells mainly enriched folate biosynthesis and linoleic acid metabolism, which was consistent with the function and activity of CD8^+^ T cells, and the CD4^+^ T cells were mainly enriched in the OXPHOS and purine metabolism pathways (Figure 4F). In addition, compared to Ths, Tregs exhibited up‐regulation of pentose phosphate pathway (GSEA, *p* < 0.001) in addition to OXPHOS (GSEA, *p* < 0.001) (Figure 4G). This finding appears to contradict previous studies showing that among immune cells derived from healthy mice not bearing tumors. These results suggest that enhanced mitochondrial oxidative metabolism is a universal metabolic feature of these T cell subtypes in different contexts.

### 2.4 Crucial factors and regulation networks governing T cell differentiation

**FIGURE 5:**
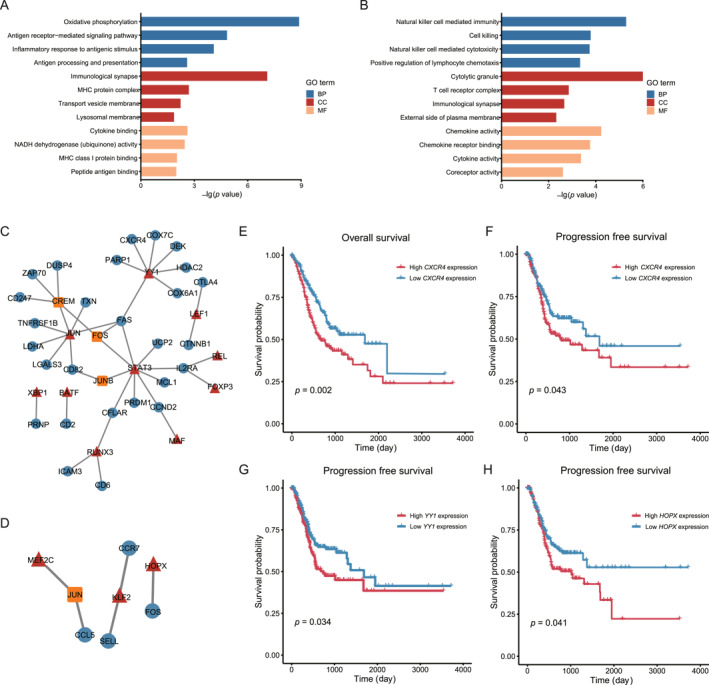
Transcriptional regulations of genes associated with T cells differentiation. (A) The results of enrichment analysis for genes that are upregulated in relation to the differentiation of T/CD4^+^ cells. (B) The findings from the enrichment analysis concerning genes that are upregulated in connection with the differentiation of T/CD8^+^ cells. (C) It presents a depiction of the transcriptional regulatory network pertinent to T/CD4^+^ cells differentiation, highlighting the transcription factors alongside their respective target genes. (D) It presents the equivalent transcriptional regulatory network, focusing on genes related to T/CD8^+^ cells differentiation. (E) Survival curves for OS were analyzed based on the stratification of patients into high and low *CXCR4* gene expression groups. (F–H) Additionally, progression free survival curves were compared between patients exhibiting high versus low expression levels of the *CXCR4*, *HOPX*, and *YY1* genes. BP, biological process; CC, cellular component; MF, molecular function; OS, overall survival.

T cells within tumor tissues exhibit unique metabolic and physiological characteristics that are shaped by the TME. To investigate the impact of genes involved in T cell differentiation on cellular physiological functions, Pearson correlation analysis was used to identify genes associated with the T cell differentiation process. We identified 570 and 197 genes that were associated with CD4^+^ and CD8^+^ T cell differentiation, respectively. In the functional analysis, we found the genes positively associated with CD4^+^ T cell differentiation were enriched in antigen processing and presentation, major histocompatibility complex protein and function related to antigen processing (Figure 5A), and CD8^+^ T cell differentiation genes were mainly involved in the cell killing, cytokine activity, and T cell receptor complex (Figure 5B). These findings were consistent with the cell function of CD4^+^ T cells and CD8^+^ T cells.

To further investigate the transcriptional regulation mechanism involved in T cell differentiation, we focused on the transcription factors (TFs) that regulate the expression of specific genes. Utilizing the TFs collected in AnimalTFDB and the transcriptional regulatory relationships in the TRRUST database, we constructed specific transcriptional regulatory networks associated with T/CD4^+^ and T/CD8+ differentiation, respectively. Analysis of the transcriptional regulatory networks revealed a total of 13 TFs in the CD4^+^ T cell network and 11 TFs in the CD8^+^ T cell network, as depicted in Figure 5C,D. We further found high expression of *CXCR4* gene is associated with worse overall survival (OS) and progression free survival (PFS) in GC patients, and the expression of its regulator *YY1* was also unfavorable on patient’s PFS (Figure 5E–G). Many studies have reported that *YY1* is an important TF involved in cell proliferation, development, DNA damage responses, and carcinogenesis, which can enhance gastric tumor cell proliferation and is correlated with poor prognosis. The *YY1* gene could also positively regulate the checkpoint receptors *PD1*, *LAG3*, and *TIM3*, and serve as a master regulator of T cell exhaustion, which is critical for immunotherapy. Additionally, the high expression of the *HOPX* gene associated with CD8^+^ T cell differentiation and could predict better PFS (Figure 5H). In summary, these findings suggest that the key genes driving T cell differentiation may be involved in important immune response regulation processes and could serve as potential immune therapeutic targets.

### 2.5 T cell differentiation‐associated biomarker signature for GC prognosis

**FIGURE 6:**
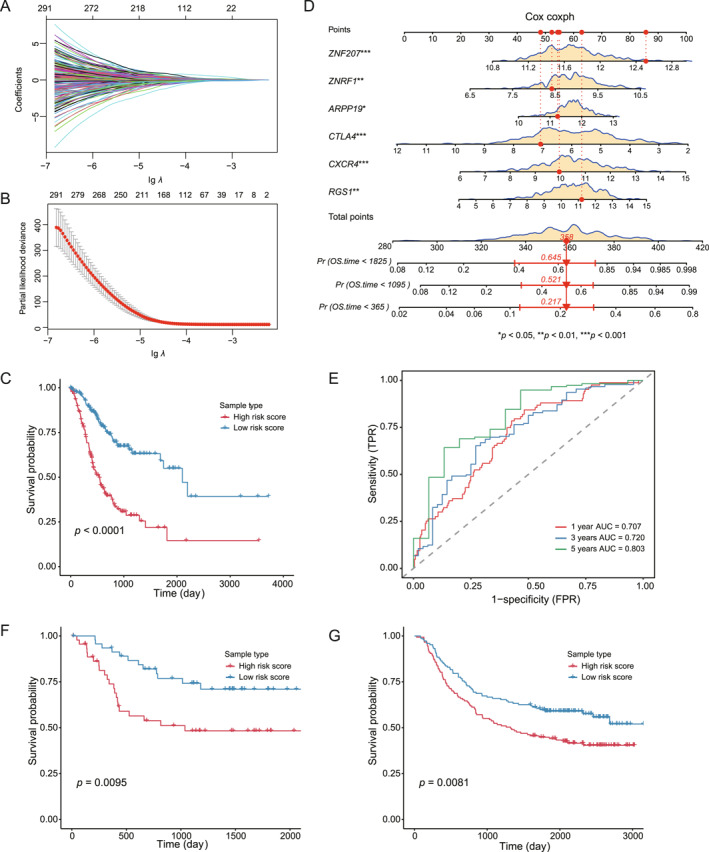
Identification and clinical validation of a prognostic signature for risk stratification in GC. (A) The dynamic progression of feature selection through LASSO‐Cox regression is illustrated. Each line represents a specific gene, demonstrating the variations in its coefficient as the value of *λ* fluctuates. The L1‐norm indicates the cumulative sum of the absolute values of non‐zero coefficients. (B) Regularization paths are shown, highlighting the changes in LASSO coefficients as *λ* is adjusted. (C) Kaplan–Meier survival curves are presented, contrasting high‐risk and low‐risk GC cohorts derived from the The Cancer Genome Atlas Program dataset. The statistical significance was evaluated utilizing the log‐rank test. (D) A nomogram is provided to predict survival risks at 1, 3, and 5 years based on six identified prognostic genes. (E) Receiver operating characteristic curves assess the predictive accuracy of the risk model at 1, 3, and 5 years intervals. (F, G) Kaplan–Meier survival analyses of GC patients stratified by risk in the GSE26899 and GSE62254 cohorts, respectively, with significance determined by the log‐rank test. FPR, false positive rate; GC, gastric cancer; LASSO, least absolute shrinkage and selection operator; OS, overall survival; TPR, true positive rate.

As an essential component of the TME, the behavior of T cells can profoundly affect tumor development and metastasis at both the molecular and cellular levels. Genes associated with T cell differentiation may serve as prognostic indicators for patient survival in GC. Therefore, we initially identified genes linked to the developmental pathway, followed by the application of LASSO regression to isolate genes that exhibit a strong correlation with patient prognosis in the The Cancer Genome Atlas Program (TCGA) dataset (refer to Figure 6A,B), resulting in the identification of six pivotal genes. Subsequently, we established a risk score model based on these genes utilizing a multivariate Cox regression analysis. This risk score effectively stratifies patients into high‐risk and low‐risk groups, revealing significantly different survival outcomes (*p* < 0.05, as illustrated in Figure 6C). Additionally, nomograms were employed to estimate the likelihood of mortality at 1, 3, and 5 years (Figure 6D). To further evaluate the predictive capability of the risk score model, the time‐dependent receiver operating characteristic (ROC) curve demonstrated a superior accuracy of the nomogram in forecasting survival, with area under curve values of 0.707, 0.720, and 0.803 for OS at 1, 3, and 5 years, respectively (Figure 6E). Furthermore, we developed an additional risk score model using data obtained from the Gene Expression Omnibus (GEO) database, which indicated that the high‐risk group was significantly associated with decreased survival in GC patients, corroborating our previous predictive findings (Figure 6F,G). In conclusion, our research highlights the potential of *RGS1*, *CXCR4*, *CTLA4*, *ARPP19*, *ZNRF1*, and *ZNF207* as reliable biomarkers for anticipating survival duration in GC.

### 2.6 Assessment of mRNA and protein expression levels in GC

**FIGURE 7:**
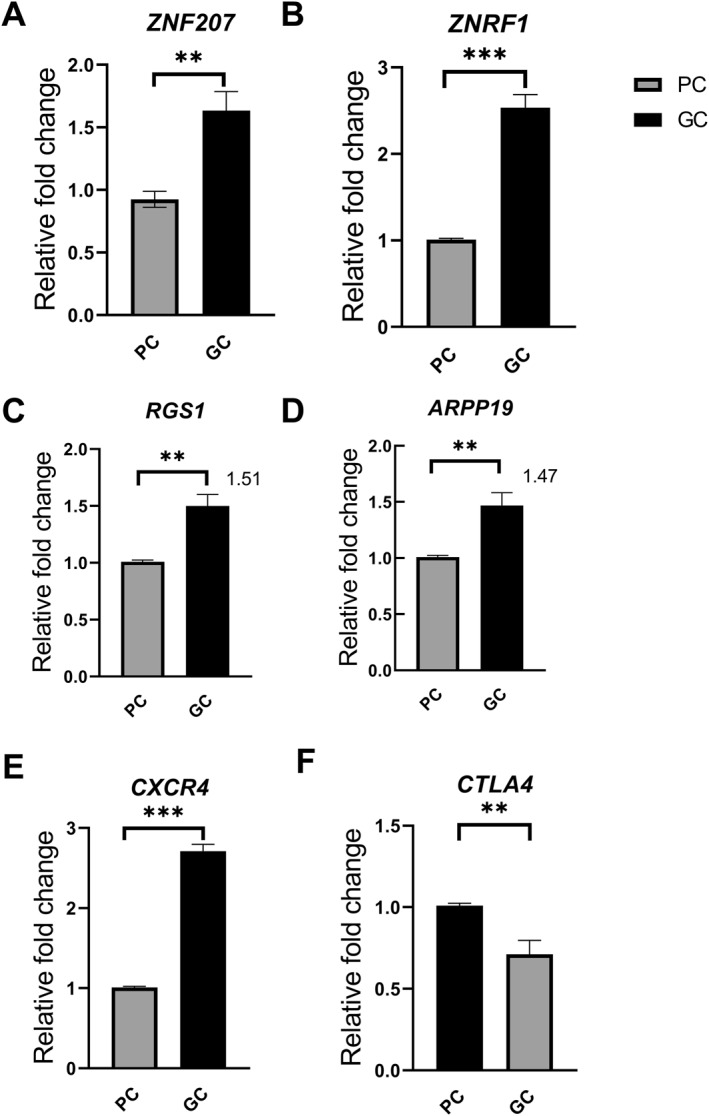
RT‐qPCR showing the mRNA expression of (A) *ZNF207*, (B) *ZNRF1*, (C) *RGS1*, (D) *ARPP19*, (E) *CXCR4*, and (F) *CTLA4* in paracancerous (principal component, PC) tissues (*n* = 10) and gastric cancer (GC) samples (*n* = 10). *p < 0.05, **p < 0.01, ***p < 0.001.

**FIGURE 8:**
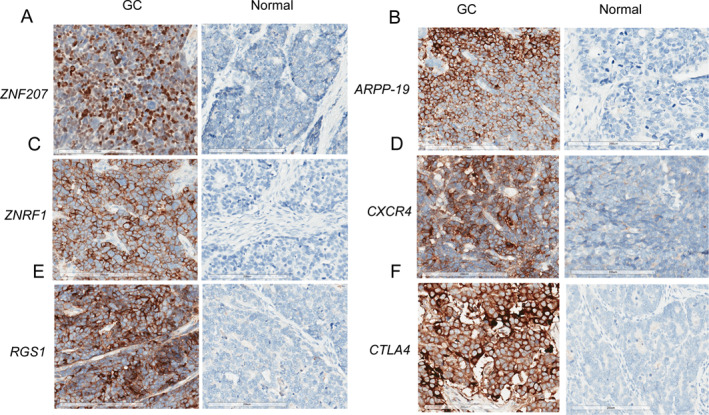
Immunohistochemical staining of target genes (A) *ZNF207*, (B) *ARPP19*, (C) *ZNRF1*, (D) *CXCR4*, (E) *RGS1*, and (F) *CTLA4* expression profiles in gastric cancer specimens and their corresponding adjacent normal gastric tissues. Brown chromogenic staining reveals positive protein expression. Blue counterstaining visualizes cell nuclei. Scale bar: 100 μm.

To further elucidate the expression patterns of six candidate genes in paracancerous tissues and GC tissues, we collected 10 pairs of gastric tumor samples and their corresponding paracancerous tissues. As illustrated in Figure 7A–F, the expression levels of *ZNF207*, *ZNRF1*, *RGS1*, *ARPP19*, and *CXCR4* were significantly elevated in GC tissues compared to paracancerous tissues, while *CTLA4* expression was decreased. Additionally, the protein expression levels of ZNF207, ZNRF1, RGS1, ARPP19, CXCR4, and CTLA4 in GC tissues and paracancerous tissues were analyzed using IHC (Figure 8A–F). Each panel displays a comparative view with GC tissues on the left, showing significant brown staining, indicative of protein presence, whereas the paracancerous tissues on the right exhibit minimal staining. These findings are consistent with the gene expression patterns observed in the IHC analysis and align with our prognostic model. These results suggest that RGS1, CXCR4, CTLA4, ARPP19, ZNRF1, and ZNF207 can serve as robust markers for predicting survival outcomes in GC patients.

## 3 DISCUSSION

In our current study, we elucidated the biological information in the scRNA‐seq profiles of GC. By clustering the cells from 23 tumor tissues of GC, we revealed the metabolic characteristics of malignant epithelial cells and the T cells in the TME. Metabolic analysis revealed that the OXPHOS pathway significantly contributes to the heterogeneity observed across various cell types. Furthermore, we pinpointed crucial genes governing T cell differentiation. Ultimately, we developed a risk score model (including *RGS1*, *CXCR4*, *CTLA4*, *ARPP19*, *ZNRF1*, and *ZNF207*) to predict the survival of GC patients.

Within the TME of GC, there were not only malignant tumor cells, but also the stromal and immune cells surrounding the tumor cells, such as macrophages, fibroblasts, T cells, plasma cells and others [17, 18]. The proliferation of tumor cells and the activity of immune cells are both closely linked to energy generation and metabolic signaling [19]. Although numerous studies have explored the single‐cell profiles of immune and tumor cells in GC, research focusing specifically on the metabolic changes within these cell types remains limited [20, 21, 22]. Therefore, in this study we characterized the metabolic characteristics of malignant cells and T cell subtypes through a weighted relative pathway activity algorithm.

Our single‐cell analysis revealed that Tregs exhibited elevated OXPHOS scores compared with cytotoxic CD8^+^ T cells, consistent with a more suppressive metabolic phenotype. This observation aligns with recent findings in other gastrointestinal tumors, where Treg metabolic adaptation supports immune evasion [23]. These results underscore the interplay between immune cell subsets and tumor metabolic states.

Tumor formation arises when normal cells proliferate in an uncontrolled manner due to a breakdown in cell cycle control. These malignant cells reengineer metabolic processes to fuel their unrestrained growth and proliferation, which provides a potential avenue for diagnosis and treatment of tumors. In our study we aimed to characterize the metabolic activity of heterogeneous cell populations within the tumors and T cell differentiation subpopulations. Our findings suggest that the OXPHOS process plays a crucial role not only in the proliferation of cancer cells but also in the differentiation of T cells. Moreover, we observed that each tumor subpopulation has its own unique metabolic pathway exhibiting high activity, implying that these metabolic differences could be a pivotal factor contributing to tumor heterogeneity. We also found that the activities of glycolysis and phenylalanine metabolism are higher in Tregs cells than Ths cells, which could provide new insights for the design of anti‐tumor drugs. Additionally, we developed a risk score model based on genes related to T cell differentiation. This model may serve as a prognostic marker for patients with GC, possibly due to the remodeling of T cell molecular mechanisms that influence tumor proliferation and metastasis. Additional investigations are required to confirm our results and to examine potential therapeutic strategies aimed at the metabolic pathways we have identified.

We acknowledge several limitations in our study: (1) sample size constraints from single‐cell data derived from a limited patient cohort; (2) the moderate predictive performance (AUC: 0.70–0.80) indicates the need for validation in larger, independent cohorts; (3) mechanistic validation through functional experiments is required to establish causative relationships between metabolic changes and immune dysfunction.

In summary, this study provided a comprehensive single‐cell analysis of the metabolic features of malignant cells and the TME. Key molecules involved in T cell differentiation were identified and their associations with patient survival were evaluated. These findings contribute valuable insights into the mechanisms driving GC progression and may inform the development of new therapeutic strategies.

## 4 MATERIALS AND METHODS

### 4.1 Data collection

The dataset pertaining to scRNA‐seq for GC was sourced from the GEO database [24], identified by the accession number GSE150290 [25]. Detailed information regarding the single‐cell sequencing samples used in this study is provided in Table S1. These data had undergone pre‐processing utilizing CellRanger software as provided by 10× Genomics. We focused exclusively on the 23 tumor tissue samples to construct expression profiles, ultimately including a total of 35,633 cells for analysis. The gene set associated with metabolic pathways was obtained from the Molecular Signatures Database (MSigDB) [26]. To further enrich our analysis, we collected bulk RNA sequencing data along with clinical details pertaining to GC patients from the UCSC Xena platform [27]. Additionally, two microarray datasets, (GSE26899 [28] and GSE62254 [29]) together with their corresponding survival data, were obtained from the GEO database to validate the prognostic significance of the identified biomarkers.

### 4.2 Single‐cell quality control and data pre‐processing

For the scRNA‐seq data, we employed the Seurat package (version 4.3.0) [30] to preprocess the data for removal of low‐quality cells, following these criteria: (1) The standard deviation for all genes within each cell is below 1; (2) There were no unique molecular identifier (UMI) counts recorded for 90% of the genes; (3) 10% or more of the expression derived from the mitochondrial or hemoglobin genes; or (4) UMI values below 100 or exceeding 20,000 [31]. This procedure resulted in the acquisition of roughly 35,633 cells for subsequent examination. In order to remove doublets, we utilized the DoubletFinder approach [32]. This method estimates the occurrence of doublets by assessing the closeness of each genuine cell within the gene‐expression landscape to synthetic doublets, which are constructed by averaging the transcriptional profiles of randomly selected pairs of cells. Upon identification of doublet cells, these were subsequently excluded from the data. To adjust for differences in sequencing depth, we normalized the count data with the LogNormalize function (Seurat’s normalization method). The top 3000 variable genes were then selected using FindVariableFeatures under default settings. We conducted principal component analysis on these genes and retained the first 20 principal components to capture the bulk of variance. Finally, cell clustering was achieved via FindNeighbors and FindClusters at a resolution of 0.8. Cell clusters were initially annotated automatically using singleR package [33], with subsequent manual refinements based on marker genes identified from the published literature and CellMarker database [34].

### 4.3 Identification of malignant epithelial cells

To identify the malignant cells within the epithelial cells population, we first reclustered all cells previously annotated as epithelial cells. We first employed the inferCNV (version 1.6.0) R package, part of the Trinity Computational Tumor Analysis Toolkit Project, to identify malignant cells through the inference of chromosomal CNVs derived from gene expression data [35, 36]. The T cells and B cells were utilized as normal reference cells to estimate CNVs within the potential tumor cell population. A gene ordering file from the human GRCh38 assembly containing each gene’s chromosomal start and end positions was created as the input of the “gene_order_file” parameter. The raw count matrix and annotation file were input to run inferCNV with cutoff = 0.1. Normal epithelial cells were subsequently delineated from cancer cells based on the evaluation of iterative clustering patterns and the calculated CNV scores [37].

### 4.4 Evaluation of intra‐tissue metabolic heterogeneity

To elucidate the intratumoral metabolic heterogeneity, the weighted relative pathway activity algorithm was employed to discern disparities in metabolic activities [38]. The initial assessment of metabolic gene expression levels within each cellular cluster was conducted by calculating the ratio of the average expression value of cells belonging to a designated cluster to the overall mean expression value across all cells. Subsequently, the weighted average of the relative expression of all genes within a particular pathway was considered the pathway’s activity score. To minimize the redundancy caused by gene overlap among various metabolic pathways, each gene was weighted by the reciprocal of the number of pathways in which it appeared. Furthermore, to mitigate the influence of sequencing quality on pathway activity scores, genes with low expression levels or high deletion rates in pathways were excluded. Specifically, lowly expressed genes were defined as those whose mRNA or protein expression levels were less than 20% of the average expression level of all genes in the pathway, whereas high deletion‐rate genes were defined as those exhibiting frequent loss‐of‐function mutations or CNVs, with a deletion rate exceeding 15% of the average deletion rate for all genes in the pathway. This exclusion ensured that the pathway activity score remained unaffected by such variables. To create a null distribution for the pathway activity scores, the cell type labels were randomly reassigned 2000 times. This methodology facilitated the assessment of the statistical significance associated with metabolic pathway activity scores for each distinct cell cluster. Consequently, a *p*‐value was computed to determine whether the pathway’s activity was significantly higher or lower than the average.

### 4.5 Trajectory analysis of T cells

Following established definitions of T cell characteristics, CD4^+^ (Th/Treg) and CD8^+^ T cells were distinguished within the initial T cell cluster analysis [39, 40]. To deduce the developmental connections, a pseudotime analysis was conducted utilizing Monocle2 [41]. Specifically, the top 2000 highly variable genes within the T cell population were selected for cell ordering using the “orderCells” function. The DDRTree dimensionality reduction algorithm was employed to project cells into a 2‐dimensional pseudotime space with max_components set to 2. Branch point identification was performed using the “Branch Expression Analysis Modeling” function with a significance threshold of *p* < 0.01 based on 1000 permutation tests. This approach identified multiple branches and nodes, where cells along each branch were presumed to represent a relatively homogeneous cell population [42].

### 4.6 Construction of transcriptional regulatory network

To identify genes correlated with T cell differentiation, we calculated Pearson correlation coefficients between gene expression profiles and quantified developmental trajectory scores of CD4^+^/CD8^+^ T cell subsets. Genes meeting significance thresholds (Pearson correlation coefficients |*r*| > 0.2, *p* < 0.05) were designated as T cell developmental process‐associated genes. We subsequently constructed transcriptional regulatory networks using TRRUST [43] and Open Repository of Transcriptional Interactions [44] databases to elucidate TF‐target gene interactions governing T cell development dynamics.

### 4.7 Development of a risk assessment model utilizing genes associated with T cell differentiation

We initiated our analysis by performing univariate analysis on T cell development‐related genes within TCGA GC dataset to identify survival‐associated candidates. Following this, the identified genes were analyzed using a penalized multivariate Cox proportional hazards survival model, utilizing an L1‐penalized estimation algorithm, commonly known as Least Absolute Shrinkage and Selection Operator, to ensure reliable selection of variables. A multivariate Cox regression model was then constructed, and genes with a *p*‐value less than 0.05 were retained to establish a risk prediction model and facilitate nomogram analysis. The ability of the model to differentiate between positive and negative prognostic outcomes was analyzed utilizing Kaplan–Meier curves. Simultaneously, its predictive accuracy over time was examined through the application of ROC curve analysis.

### 4.8 Quantitative real‐time polymerase chain reaction

Total RNA was extracted with VeZol Reagent and assessed for quality using NanoDrop 2000 and agarose gel electrophoresis. First‐strand cDNA was synthesized from 1 μg of RNA using the PrimeScript RT Master Mix (Takara), according to the manufacturer’s protocol. Quantitative polymerase chain reaction (PCR) was performed on the Thermo Fisher 7500 Fast Real‐Time PCR System utilizing TB Green Premix Ex Taq II (Takara) in a 10 μL reaction, with gene‐specific primers and 1 μL cDNA. The cycling conditions were: initial denaturation at 95°C for 30 s, followed by 40 cycles of 95°C for 5 s and 60°C for 30 s, with subsequent dissociation curve analysis. Primer sequences for glyceraldehyde‐3‐phosphate dehydrogenase (GAPDH) and target genes used in the qRT‐PCR analysis are listed in Table 1.

**TABLE 1:** Primer sequences for RT‐qPCR validation of target genes and endogenous controls.

Gene name	Primer sequences (5′–3′)	Optimum temperature (°C)	Product size (bp)
*GAPDH*	F: GGGAAACTGTGGCGTGAT	60	299
	R: GAGTGGGTGTCGCTGTTGA		
*RGS1*	F: TTGTGCATTCAGATGCTGCTAAAC	60	155
	R: GAGGAACCTGGGATAAGAGTCC		
*CXCR4*	F: CTCCTCTTTGTCATCACGCTTCC	60	127
	R: GGATGAGGACACTGCTGTAGAG		
*CTLA4*	F: ACGGGACTCTACATCTGCAAGG	60	121
	R: GGAGGAAGTCAGAATCTGGGCA		
*ARPP19*	F: CAAAAGCCTGGAGGTTCAGATTTC	60	150
	R: GTCACCAGTGACCTCCGTCTTA		
*ZNRF1*	F: CCATAGAGACGGGATGCTGTAC	60	129
	R: GCCACAGACTTGGAGCAAATGG		
*ZNF207*	F: ATGATGCCACCTGGACCAGGAA	60	116
	R: GCTGAAACAGCCTGTGCTTGAG		

### 4.9 Immunohistochemistry

GC samples were initially fixed and subsequently incubated at 65°C for 3 h. After fixation, the samples underwent processes of dewaxing and antigen retrieval. To reduce nonspecific binding, tissue sections were blocked with 5% bovine serum albumin for 1 h at room temperature. Immunohistochemical staining was then carried out using primary antibodies targeting RGS1 (Sigma, HPA028453), CXCR4 (Cell Signaling Technology, #97680), CTLA4 (Cell Signaling Technology, #53560), ARPP19 (Proteintech, 11678‐1‐AP), ZNRF1 (LSBio, LS‐C809970), and ZNF207 (Invitrogen, PA5‐53535). Following incubation with primary antibodies, sections were treated with a biotinylated goat anti‐rabbit secondary antibody for 1 h at room temperature. Detection was performed using diaminobenzidine substrate, and stained sections were visualized and imaged with a Nikon Eclipse Ci microscope equipped with a 20×/0.75 NA objective.

### 4.10 Statistical analysis

Statistical analyses and graphical outputs were generated using R statistical software (version 4.2.0). Intergroup comparisons were conducted using the Wilcoxon rank‐sum test. Survival probabilities were estimated via the Kaplan–Meier method, and differences between survival curves were evaluated using the log‐rank test. A significance level of *p* < 0.05 was adopted, with appropriate corrections for multiple testing implemented.

## AUTHOR CONTRIBUTIONS

**Junjun Liu**: Conceptualization, methodology, investigation, formal analysis, writing—original draft. **Rui Zhao**: Data curation, validation. **Guodong Yao**: Investigation, resources. **Zhao Liu**: Investigation, resources. **Runze Shi**: Investigation. **Jingshu Geng**: Formal analysis, visualization. **Guanying Liang**: Conceptualization, methodology, supervision, writing—review and editing, funding acquisition. **Kexin Chen**: Conceptualization, supervision, project administration, writing—review and editing, funding acquisition.

## CONFLICT OF INTEREST STATEMENT

The authors declare no conflicts of interest.

## ETHICS STATEMENT

This study was approved by the Medical Ethics Committee of Harbin Medical University Cancer Hospital and conducted in accordance with the Declaration of Helsinki. Written informed consent was obtained from all patients who provided tissue samples. All publicly available datasets used in this study were obtained from databases with appropriate ethical approvals.

## Supporting information

Table S1

## Data Availability

The scRNA‐seq data (GSE150290), microarray datasets (GSE26899, GSE62254), and bulk RNA‐seq data with clinical information were obtained from public databases (GEO and UCSC Xena). Metabolic gene sets were sourced from MSigDB. Raw RT‐qPCR and IHC data are available upon reasonable request from the corresponding authors. All data usage complies with database terms and institutional guidelines.
