# Supplementary material for: Metabolic‐immune interactions in gastric cancer T cells: A single‐cell atlas for prognostic biomarker identification
Source: Quant Biol. 2026 Jan 1;14(2):e70027. doi: 10.1002/qub2.70027 (PMC12806102; doi:10.1002/qub2.70027)
Supplement: Supplementary file 1 — Table S1 [file QUB2-14-e70027-s001.docx]

**Supplementary Tables**

**Table S1: The information of samples used in this study of single-cell dataset.**

| Sequencing technology | Sample | Cell/sample number | Tissue | Sex | Diagnosis |
| --- | --- | --- | --- | --- | --- |
| Single-cell | GSM4546301 | 1316 | Tumor | Male | EGC |
|  | GSM4546303 | 1947 | Tumor | Male | AGC |
|  | GSM4546305 | 1200 | Tumor | Female | EGC |
|  | GSM4546307 | 2989 | Tumor | Female | EGC |
|  | GSM4546309 | 1920 | Tumor | Male | EGC |
|  | GSM4546311 | 1853 | Tumor | Male | EGC |
|  | GSM4546313 | 1111 | Tumor | Male | EGC |
|  | GSM4546315 | 2107 | Tumor | Female | EGC |
|  | GSM4546317 | 1983 | Tumor | Male | EGC |
|  | GSM4546319 | 658 | Tumor | Male | EGC |
|  | GSM4546321 | 2068 | Tumor | Female | AGC |
|  | GSM4546323 | 1606 | Tumor | Female | AGC |
|  | GSM4546325 | 1071 | Tumor | Male | EGC |
|  | GSM4546327 | 1676 | Tumor | Female | AGC |
|  | GSM4546329 | 1256 | Tumor | Male | AGC |
|  | GSM4546331 | 992 | Tumor | Male | EGC |
|  | GSM4546333 | 967 | Tumor | Male | EGC |
|  | GSM4546335 | 551 | Tumor | Male | EGC |
|  | GSM4546337 | 1445 | Tumor | Male | EGC |
|  | GSM4546339 | 2104 | Tumor | Female | AGC |
|  | GSM4546342 | 1466 | Tumor | Male | EGC |
|  | GSM4546344 | 2049 | Tumor | Male | AGC |
|  | GSM4546346 | 1298 | Tumor | Female | AGC |

EGC: Early gastric cancer; AGC: Advanced gastric cancer
